# ASSOCIATIONS AMONG LATENT FACTORS OF METABOLIC LOAD, BRAIN VOLUME, AND COGNITION IN HEALTHY AGING

**DOI:** 10.1093/geroni/igac059.006

**Published:** 2022-12-20

**Authors:** Sandra Duezel, Johanna Drewelies, Ulman Lindenberger, Elisabeth Steinhagen-Thiessen, Ilja Demuth, Simone Kühn

**Affiliations:** Max-Planck-Insitute for human developement, Berlin, Berlin, Germany; Humboldt University Berlin, Berlin, Berlin, Germany; Max Planck Institute for Human Development, Berlin, Berlin, Germany; Charité - Universitätsmedizin Berlin, Berlin, Berlin, Germany; Charité Universitätsmedizin Berlin, Berlin, Berlin, Germany; Max Planck Institute for Human Development, Berlin, Berlin, Germany

## Abstract

Metabolic syndrome (MetS) refers to risk factors for cardiovascular disease associated with reduced physical fitness, higher disease burden, and impaired cognitive functions. Available evidence indicates that metabolic functioning is related to brain structure at the level of specific indicators, such as hypertension and voxel-based morphometry, respectively. However, attempts to relate metabolic load to brain morphology at the construct level using structural equation modeling are scarce. Here, we used confirmatory factor analysis to: (a) examine level and change associations among latent factors of metabolic risk, regional grey-matter integrity (GMI), and cognition; (b) test whether these associations differ by sex. Analyses were based on a sample of 1,100 healthy adults (52% female) aged 60 to 88 years from the Berlin Aging Study II, and included MRI data for a sub sample of 341 (37 % female) individuals. Metabolic risk was defined by waist circumference, triglycerides, fasting blood glucose, and high-density lipoprotein; regional GMI by mean diffusivity, magnetization ratio transfer ratio, and VBM-based volume estimates; and cognition, represented by the three latent constructs episodic memory, working memory and fluid intelligence. Initial analyses indicate that individuals with lower metabolic risk show greater GMI in prefrontal cortex. Also, we found that greater prefrontal GMI was associated with higher fluid intelligence and working memory in men only. Additionally, we investigated the effects of previous levels of metabolic risk and cognition on subsequent changes in both domains over time. We highlight the benefits of latent factors for establishing brain-behavior relations, and discuss putative physiological substrates of individual differences in GMI.

